# Chimeric blood vessels sustained development of the xenogeneic antler: a unique model for xenogeneic organ generation

**DOI:** 10.1093/lifemedi/lnac021

**Published:** 2022-07-09

**Authors:** Datao Wang, Xin Wang, Hengxing Ba, Jing Ren, Zhen Wang, Hai-Xi Sun, Liang Chen, Chuanyu Liu, Yusu Wang, Jiping Li, Longqi Liu, Tianbin Liu, Yunzhi Peter Yang, Guang-Hui Liu, Ying Gu, Chunyi Li

**Affiliations:** Institute of Antler Science and Product Technology, Changchun Sci-Tech University, Changchun 130600, China; Institute of Special Wild Economic Animals and Plants, Chinese Academy of Agricultural Sciences, Changchun 130112, China; BGI-Shenzhen, Shenzhen 518083, China; Hubei Key Laboratory of Cell Homeostasis, RNA Institute, College of Life Sciences, Wuhan University, Wuhan 430072, China; Institute of Antler Science and Product Technology, Changchun Sci-Tech University, Changchun 130600, China; College of Chinese Medicinal Materials, Jilin Agricultural University, Changchun 130118, China; Institute of Antler Science and Product Technology, Changchun Sci-Tech University, Changchun 130600, China; College of Chinese Medicinal Materials, Jilin Agricultural University, Changchun 130118, China; Institute of Antler Science and Product Technology, Changchun Sci-Tech University, Changchun 130600, China; BGI-Shenzhen, Shenzhen 518083, China; Guangdong Provincial Key Laboratory of Genome Read and Write, BGI-Shenzhen, Shenzhen 518120, China; Hubei Key Laboratory of Cell Homeostasis, RNA Institute, College of Life Sciences, Wuhan University, Wuhan 430072, China; BGI-Shenzhen, Shenzhen 518083, China; Institute of Antler Science and Product Technology, Changchun Sci-Tech University, Changchun 130600, China; Jilin Provincial Key Laboratory of Deer Antler Biology, Changchun 130600, China; Institute of Antler Science and Product Technology, Changchun Sci-Tech University, Changchun 130600, China; Jilin Provincial Key Laboratory of Deer Antler Biology, Changchun 130600, China; BGI-Shenzhen, Shenzhen 518083, China; BGI-Shenzhen, Shenzhen 518083, China; College of Life Sciences, University of Chinese Academy of Sciences, Beijing 100049, China; Department of Orthopaedic Surgery, Stanford University, Stanford, CA 94304, USA; College of Life Sciences, University of Chinese Academy of Sciences, Beijing 100049, China; Institute for Stem Cell and Regeneration, Chinese Academy of Sciences, Beijing 100101, China; State Key Laboratory of Membrane Biology, Institute of Zoology, Chinese Academy of Sciences, Beijing 100101, China; BGI-Shenzhen, Shenzhen 518083, China; Guangdong Provincial Key Laboratory of Genome Read and Write, BGI-Shenzhen, Shenzhen 518120, China; College of Life Sciences, University of Chinese Academy of Sciences, Beijing 100049, China; Institute of Antler Science and Product Technology, Changchun Sci-Tech University, Changchun 130600, China; College of Chinese Medicinal Materials, Jilin Agricultural University, Changchun 130118, China; Jilin Provincial Key Laboratory of Deer Antler Biology, Changchun 130600, China

**Dear editor,**

Xenogeneic organs are expected to provide a solution to the problems of organ shortage and immune rejection. Rashid et al. [1] proposed using genetically engineered “organ niches” in large animals (blastocyst complementation) to generate xenogeneic organs from pluripotent stem cells. However, there are formidable technical challenges and ethical concerns over gamete and neural contribution of human cells. Therefore, transplanting stem cells or tissue into animals rather than blastocysts is much less controversial. In this case, immunodeficient animals would have to be used for the generation of xenogeneic organs. Along this line, Oldani et al. [2] created chimeric livers by injecting rat hepatocytes into immunodeficient mice to generate liver, and then transplanted the livers back into rats. Kaneko et al. [3] obtained functional sperm of pig through transplanted pig testicular tissue into nude mice. We subcutaneously transplanted antlerogenic periosteum (AP, periosteum overlies the deer frontal crest, Fig. S1) into the nude mouse and found almost 100% grafted AP formed xenogeneic antlers [4]. Given such a high successful rate and relatively simple surgery, we recognized the value of xenogeneic antler model for the investigation of the interactions between the grafted tissue/cells and the host milieu, and cellular origin (i.e. from transplants or the host) of the xenogeneic organ.

In the present study, we mapped the composition of cell types in the xenogeneic antlers paying special attention to their origin (deer or mouse) using single-cell RNA sequencing (scRNA-seq) and explored the interactions between the grafts and the host cells. Here we collected the xenogeneic antlers on days 14, 21, and 35 after grafting surgery (termed as DM14, DM21, and DM35) for scRNA-seq. The scRNA-seq data of the dormant AP (DAP) and the activated AP (AAP) in deer (in submission) were incorporated into the data of the present study to enable a comparative analysis (Figs. 1A and S2A). After quality control and integration of the five sets of scRNA-seq data, we obtained a total of 27,167 cells. In total, 11 types of cells were identified through the marker genes, including antler stem cells (ASCs), progenitor cells, chondrocytes, mural cells, stem/mural-like cells, lymphatic endothelial cells, vascular endothelial cells (VECs), monocytes/macrophages, mast cells and natural killer/T cells, and hematopoietic stem cells (HSCs) (Figs. 1B, 1C and S2B–D).

**Figure 1:**
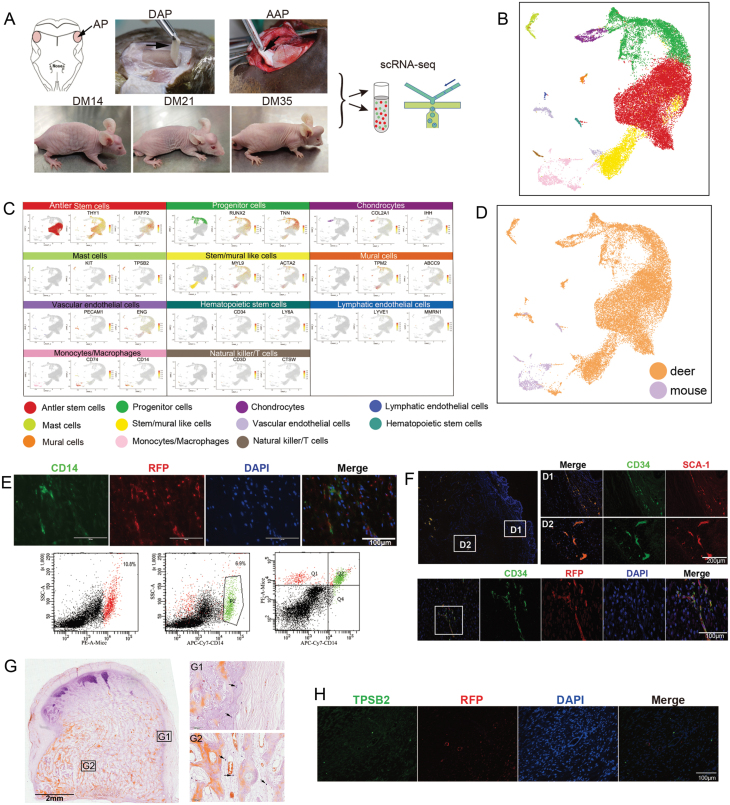
scRNA-seq profiling of the cell composition of the xenogeneic antlers (DMs). (A) Experimental design (mixture of schematic drawing and photos). (B) UMAP diagram showing different cell types across the different tissue samples (DAP, AAP, DM14, DM21, and DM35). (C) UMAP diagram to show the expression status of marker genes corresponding to each cell type. The first column shows the cell type, and the second and third columns show the expression status of the corresponding marker genes. (D) UMAP image of cell composition to show the cell origin. (E) Immunofluorescent staining and flow cytometry. Note that monocytes/macrophages were mainly mouse-derivative. Tissue sections of the DM21 collected from the red fluorescent nude mice were stained with CD14 antibody (green) and RFP (red) in immunofluorescent staining (upper); and single cells were dissociated from the fresh DM21 and stained with CD14-FITC (lower panel). (F) Tissue sections of the DM35 were stained with CD34 (green) and SCA-1 (red) for HSCs. Double staining showed that HSCs were mouse-derivative (lower panel). (G) Aldehyde fuchsin staining of DM35 tissue sections to visualize mast cells (arrows). (H) Double staining demonstrated that mast cells were deer-derivative.

We further analyzed the species origin of these cells in the DMs. The results showed that the DMs comprised mainly deer-derived cells (about 90%), and during the development of these DMs, the relative ratios of deer-derived cells and mouse-derived cells were maintained in a dynamic balance (Figs. 1D S3A and S3B). The majority of cell types showed specific characteristics of species origin: bone-lineage cells (ASCs, progenitor cells, and chondrocytes), stem/mural-like cells, mural cells, and mast cells were found to be exclusively deer-derived; monocytes/macrophages and natural killer/T cells found to be mainly mouse-derived. HSCs were present on the DM14 (i.e. day 14) and all were of mouse origin. VECs were both deer- and mouse-derived, and the proportion of mouse-derived cells gradually increased along with the growth of the DMs (Fig. S3C). To enable tracing of the cell origin, we produced xenogeneic antlers in red fluorescent nude mice (Fig. S4A).

The results of scRNA-seq showed that immune cells were mainly derived from the mice, while mast cells were exclusively from deer (Fig. S4B). Immunofluorescence and flow cytometry analysis demonstrated that monocytes/macrophages were mouse-derivative (Figs. 1E and S4C). Histological staining showed HSCs not only were located in the bone trabecular region but also subcutaneously, and they were all derived from the mouse (Fig. 1F). However, the disproportionately high number of HSCs in the DMs (13.5% of nucleated cells in DM14, 18.1% in DM21, and 12.6% in DM35) is surprising, being much higher than that of peripheral blood (0.03%–0.1% of nucleated cells), and even higher than that of bone marrow (0.33%–1.98%) [5, 6]. Histological staining showed mast cells were distributed throughout the entire xenogeneic antler, and they were all deer-derived (Figs. 1G and 1H).

Recirculation is essential for the survival and development of tissue/organ transplants. Results of scRNA-seq showed that VECs were mainly derived from the mice, while mural cells and stem/mural-like cells were all deer-derived (Fig. 2A). VECs were found to be derived from both deer and mouse in the early growth stage (DM14); however, in the stage of DM35, VECs were mainly derived from the mice. In contrast, mural cells and stem/mural-like cells were found to be derived only from deer (Fig. 2B). Immunofluorescence staining of xenogeneic antlers in red fluorescent nude mice confirmed that the mural cells (SMA^+^) were derived from the deer, and the VECs (CD31^+^) were mainly derived from the mice (Fig. 2C).

**Figure 2:**
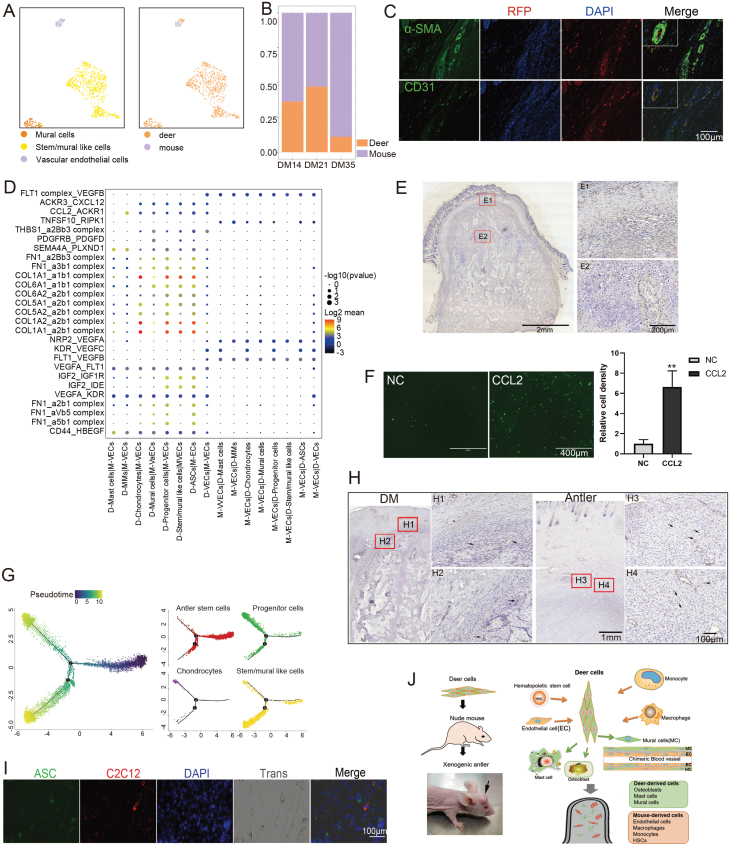
Chimeric blood vessels sustained the development of xenogeneic antler. (A) UMAP diagram of blood vessel-related cells in scRNA-seq. The left picture showed cell type and the right picture cell source. Note that mural cells, stem/mural-like cells were deer-derivative, and VECs were mainly derived from the mouse. (B) Proportions of VECs of different origin in the xenogeneic antlers (DM14, DM21, and DM35). (C) Representative immunofluorescence staining of the DMs was collected from the red fluorescent nude mice. Double immunofluorescence staining demonstrated species origin of the vascular cells; mouse cells labeled with red fluorescent protein (RFP, red). VECs stained with CD31 (green) were mainly mouse-derivative, while mural cells stained with α-SMA (green) were deer-derivative (scale bar = 100 µm). (D) Overview of selected ligand–receptor interactions between ASCs and deer-derivative cells in the DM14. The dot size indicates the level of significance and the color indicates the expression profile of the ligand–receptor pair. MMs, monocytes/macrophages; D-Mast cells, deer-derived mast cells; M-VECs, mouse-derived vascular endothelial cells. (E) Representative immunohistochemistry of CCL2 on the sections of xenogeneic antler (DM21). Note that CCL2 was not only expressed in mesenchymal cells, but also in some chondrocytes. (F) CCL2 (10 µg/mL) significantly promotes the migration of human umbilical vein endothelial cells (HUVECs). Data are shown as the mean ± SEM, *n* = 6. **P < .01 (two-tailed *t* test). (G) Bone/mural cells development of DM14 through pseudotime trajectory analysis. The left and the middle panels: trajectory diagram showed differentiation of stem cells, progenitor cells, chondrocytes, stem/mural cells, and mural cells. (H) Representative immunohistochemistry of α-SMA on the sections of both DMs and orthotopic antlers. Note that α-SMA was expressed not only by the cells that were around vessels but also by some scattered cells (arrows). (I) ASCs (green) were co-cultured with C2C12 (red) at 1:1 ratio. Note that multinucleated muscle cells consisted of both cell types (scale bar = 100 µm). (J) Graphical summary.

To explore how mouse VECs participate in the construction of blood vessels that anastomose the deer tissue and the host, we applied CellPhoneDB to reveal the interactions between VECs and deer-derived cells. The results demonstrated that the cell–cell interactions in the DMs were more extensive compared to AAP and DAP, and there were strong interactions between the mouse VECs and deer ASCs/mural cells/progenitor cells/chondrocytes (Figs. 2D S5A and S5B). GO enrichment (Version 3.12.1) of interaction pairs was found to be mainly involved in angiogenesis, cell proliferation, and migration (Fig. S5C). The classical interaction pairs that regulate angiogenesis [7], namely VEGF-FLT, VEGF-KDR, and IGF-IGFR NRP-SEMA, were identified between the deer cells and mouse VECs (Figs. 2D and S5D). The ASCs and their derived cells were found to express a high level of CCL2 (Fig. 2E), it was considered to be the key factor for recruiting endothelial cells (Fig. 2F).

Previous studies demonstrated that mural cells have multiple origins, including medial smooth muscle cells (SMCs), transdifferentiation from VECs, adventitial fibroblasts, and stem cells from circulation [8]. We focused on the origin of mural cells in the DMs and carried out a pseudotime trajectory analysis, including ASCs, progenitor cells, chondrocytes, and stem/mural-like cells. The results showed that stem cells differentiated into two branches, mural-like cells and chondrocytes, respectively (Fig. 2G). The immunohistochemistry staining demonstrated that not only cells around vessels expressed α-SMA, but so did many scattered cells (Fig. 2H). Clark et al. [9] also reported that some cells in precartilage region of the antler (not in vessels) were α-SMA positive and proposed that this is the major region of vascular formation. We demonstrated that these cells may be differentiated from the ASCs, as when co-cultured with C2C12 cells, ASCs can differentiate into muscle-like cells (Figs. 2I and S6A). At the same time, we demonstrated via EdU labeling approach that mural cells can proliferate in the DM (Fig. S6B). In so doing, deer-derived blood vessel wall cells and mouse VECs together form deer-mouse chimeric blood vessels, sustaining the development of xenogeneic antler development (Fig. S6C).

We have developed a unique xenogeneic organ model that is well suited for the study of cell origin and cell interactions between graft and host. This approach would provide information to help understanding the mechanisms underlying the production of xenogeneic organs. We mapped the composition of cell types in xenogeneic antlers through scRNA-seq paying special attention to their origin (deer or mouse).

The anastomotic blood vessels were found to be chimeric, with the endothelial cells being derived from the host and the mural cells from the AP graft. We confirmed that ASCs can differentiate into SMCs. AP tissue per se secretes multiple angiogenic factors, such as VEGF, S100A4, TMSB10, PTN, and CCL2. These angiogenic factors may well be the inducers for the migration of host endothelial cells into the vessels (mainly mural cells) that grow out of the graft, thus forming the chimeric vessels. Irrespective of the donor of an engineered organ, transplantation is followed by a narrow time window opportunity in which the host vasculature must supply the organ with blood to avoid ischemia, injury, and necrosis [10]. It is possible chimeric blood vessels quickly build up a circulatory network which is essential for the survival and development of the transplants. Unveiling the mechanism underlying this unique model of xenogeneic organ formation would assist with the design of a system comprising particularly selected components of tissue/cells for engineering to promote survival and maintenance of transplants in clinic settings.

## Research limitations

ScRNA-seq provided lots of information but little mechanistic insights. Antler is not an organ of humans, although it can be considered as a unique model. It still has remote relevance to the generation of human xenogeneic organs, especially, we don’t know enough about the regulatory mechanisms. Much work is needed to unveil the mechanism underlying xenogeneic antler growth.

## Supplementary Material

lnac021_suppl_Supplementary_Figures

## Data Availability

Single-cell sequencing data have been deposited in the China National GeneBank DataBase (https://db.cngb.org/cnsa/; CNP0002313) and Sequence Read Archive (SRA) data (https://www.ncbi.nlm.nih.gov/sra; PRJNA777734).
