# Supplementary material for: Chimeric blood vessels sustained development of the xenogeneic antler: a unique model for xenogeneic organ generation
Source: Life Med. 2022 Jul 9;2(1):lnac021. doi: 10.1093/lifemedi/lnac021 (PMC11749460; doi:10.1093/lifemedi/lnac021)
Supplement: lnac021_suppl_Supplementary_Figures [file lnac021_suppl_Supplementary_Figures.pdf]

Materials and Methods

Animals and transplantation

Nude mice (4-6 weeks old) were purchased from Changsheng Biotechnology Co. LTD (Liaoning, China). mCherry transgenic mice were purchased from Labgene Biotechnology Co. LTD (Jiangsu, China). We obtained fluorescent nude mice by crossing fluorescent mice with nude mice in our laboratory. AP were collected from 6–8-month-old male sika deer following the methods reported by Li et al. [1]. The sampled AP was divided into 0.5 x 0.5 cm pieces, which were transplanted to head of nude mice or fluorescent nude mice [2]. For autologous transplantation, xenogeneic antlers were transferred back to the same deer from the mice.

Preparation of Single-Cell Suspension

Xenogeneic antlers were collected at day 14, 21 and 35 after transplantation. After peeling off the skin, Half of the xenogeneic antlers were fixed with 4% paraformaldehyde, the other half were cut into <1 mm^3 segments. Minced tissues were washed twice with PBS, and then digested in the digestive solution containing DMEM (Sigma-Aldrich, USA), 100 µg/ml collagenase type I and 100 µg/ml type II (Invitrogen, USA) for 60-90 min at 37 °C with intermittent shaking [3]. Live cell numbers in the digest were counted using AO/PI double fluorescence staining kit (Beyotime, China). When more than 20,000 live cells were released, digestion was stopped by adding 10% (V/V) fetal bovine serum (Gibco, USA). The digests were passed through a 70-µm filter and cells were collected by centrifugation (500g for 5 min at 4 °C). To remove red blood cells, cell pellets were treated with 1× RBC lysis buffer (Beyotime) for 5 min at room temperature and washed with PBS. Live cell numbers were counted again using AO/PI double fluorescence staining kit.

Preprocessing of scRNA-seq data

Cell Ranger (version 3.0.2, 10X Genomics) was used to align the sequencing data to the sika deer genome (in house) and to obtain the expression counts matrixes. Then, the matrixes were processed by using Seurat R package (version 3.2.1) [4] and low-quality cells were filtered out. Furthermore, the expression matrixes of multiple samples were integrated by using ‘IntegrateData’ function, the first 30 principal components were selected to perform dimensionality reduction. We used the ‘FindAllMarkers’ function to identify the differential expression genes for different clusters. The cells were annotated based on the specific marker genes of the cell type. specifically, considering that there were only few studies on the cell types of sika deer, we referred to the marker genes of

other mammals.

Correlation analysis

For the analysis of the similarity between cell types, we used the AverageExpression function in Seurat R package to calculate the average expression level of each cell type, and thereby obtain the Pearson correlation coefficient.

Regulon analysis

The SCENIC (version 1.1.3) [5] was applied to predict the transcriptional factor regulation network. At first, the co-expression modules were deduced only by the expression profile. Second, to obtain the gene regulation modules (regulons), the above co-expression modules were refined by combining with Rcis Target database. Third, to indicate the network activity of each cell, the area under curve (AUC) was calculated.

Analysis of cell-cell interactions

For cell-cell interactions analysis, we used CellPhoneDB software (version 2.1.7) [6]. In details, the ligand or receptor expressed in more than 10% of the total cells were retained for further analysis. The average expression levels of each ligand-receptor pairs in the different cell types were compared, and only those with p-value < 0.05 were kept for the prediction of cell-cell interactions. The same cell types of different species origins were separated into different categories, in order to distinguish the interactions between cells from the same species to the different species.

Functional enrichment analysis

Clusterprofiler was used to perform enrichment of the GO biological process and KEGG enrichment analysis, and the entry with qvalue below 0.05 was considered to be significant. The visualization of the analysis results was achieved by using ggplot2 package.

Pseudo-time trajectory analysis

The cell trajectory analysis was carried out by using Monocle (version 2.14.0) [7]. We extracted the UMI counts matrix corresponding to the selected cell types from the Seurat object, and 'DispersionTable' function of Monocle was performed to obtain the high dispersion genes. Based on these, cells were arranged to obtain the pseudo-time trajectory. To identify the genes that play vital roles in the stem cell fate decision, branched expression analysis modeling (BEAM) was performed and the genes that were branch-dependent with q-value $< 10^{-4}$ were selected for

visualization.

Immunofluorescence and Immunohistochemistry

Paraffin-embedded tissues were sectioned, rehydrated, boiled in sodium citrate to expose antigens, endogenous peroxidase was blocked using 3% H_2O_2, and then with 5% BSA in PBS. The tissue sections were incubated with primary antibodies overnight at 4°C, washed with PBS for three times, incubated with secondary antibodies conjugated with fluorescein (for immunofluorescence) or with HRP (for immunohistochemistry) for 1 hr., and finally counterstained with DAPI (5 μg/ml) or hematoxylin, respectively. We used Gomri aldehyde fuchsin staining kit (Gefan, Shanghai) For aldehyde fuchsin staining. The rehydrated sections were directly stained following manufacturer protocols. HE and HE/Alcain blue staining were done following routine protocols. All antibodies used in this study are listed in Table S1.

Proliferating cell detection

For proliferating cell detection, EdU (5 mg/kg per d; Beyotime) were administered through intraperitoneal injection 1 day before sampling. Preparation of the tissue sections was the same as the above-described, and EdU-labeled cells were analyzed according to the standard protocol (Cell-Light EdU Apollo488 In Vitro Kit, Ribo).

Flow cytometry analysis

Xenogeneic antlers were collected from the red fluorescent nude mice, and procedure for making single-cell suspension were the same as the above-described. Cells were fixed with 4% paraformaldehyde for 30 mins. For mCerry staining, cells were stained with rabbit Anti-mCherry (Abcam, USA). Washed with PBS for three times and then stained with Goat Anti-Rabbit IgG H&L Cy3 (Abcam). For CD14 stained, cells were stained with rat anti-mouse CD14 APC-Cy7 (BD biosciences, USA). Flow cytometry analysis was performed using FACSCanto (BD biosciences), and a minimum of 10,000 cells were collected for each sample. The results were analyzed using Cellquest software (BD biosciences).

Myogenic differentiation

C2C12, a mouse skeletal muscle cell line [8], were purchased from Procell (Wuhan, China) and APCs were from our laboratory. The cultured APC and C2C12 were passaged using 0.25% trypsin respectively and separately counted. The APCs were stained with 5 μM CFSE (Invitrogen) and C2C12 stained with 2 μM PKH26 (Sigma), and thereafter the two cell types were mixed at the ratio

1:1. The mixed cells were seeded in the wells of 24-well plates at a density of 10^5/well. The medium was replaced by the induction medium (DMEM with 2% equine serum) on the next day [9]. The cells were counterstained with 10 μg/ml Hoechst and examined under a fluorescent microscope 7 days after. The expression of Myosin was analyzed using mouse anti-Myosin (Abcam).

Statistical analysis

Data are expressed as mean ± SD. Differences were analyzed by unpaired Student's t-tests. $P < 0.05$ was considered significant. Statistical analyses were performed using GraphPad Prism version 8.00 (GraphPad Software, USA) and indicated in the figure legends.

1. Li C and JM Suttie. Deer antlerogenic periosteum: a piece of postnatally retained embryonic tissue? Anat Embryol (Berl) 2001;5: 375-388.
2. Li C, X Gao, F Yang, SK Martin, SR Haines, X Deng, et al. Development of a nude mouse model for the study of antlerogenesis--mechanism of tissue interactions and ossification pathway. J Exp Zool B Mol Dev Evol 2009;2: 118-35.
3. Reichard A and K Asosingh. Best Practices for Preparing a Single Cell Suspension from Solid Tissues for Flow Cytometry. Cytometry A 2019;2: 219-226.
4. Butler A, P Hoffman, P Smibert, E Papalexi,R Satija. Integrating single-cell transcriptomic data across different conditions, technologies, and species. Nature Biotechnology 2018;5: 411-420.
5. Aibar S, CB González-Blas, T Moerman, VA Huynh-Thu, H Imrichova, G Hulselmans, et al. SCENIC: Single-cell regulatory network inference and clustering. Nature Methods 2017;11: 1083-1086.
6. Efremova M, M Vento-Tormo, SA Teichmann,R Vento-Tormo. CellPhoneDB: inferring cell-cell communication from combined expression of multi-subunit ligand-receptor complexes. Nat Protoc 2020;4: 1484-1506.
7. Trapnell C, D Cacchiarelli, J Grimsby, P Pokharel, S Li, M Morse, et al. The dynamics and regulators of cell fate decisions are revealed by pseudotemporal ordering of single cells. Nature Biotechnology 2014;4: 381-386.
8. McMahon DK, PA Anderson, R Nassar, JB Bunting, Z Saba, AE Oakeley, et al. C2C12 cells: biophysical, biochemical, and immunocytochemical properties. Am J Physiol 1994;6 Pt 1: 1795-1802.
9. Cheng CS, Y El-Abd, K Bui, YE Hyun, RH Hughes, WE Kraus, et al. Conditions that promote primary human skeletal myoblast culture and muscle differentiation in vitro. Am J Physiol Cell Physiol 2014;4: 385-395.

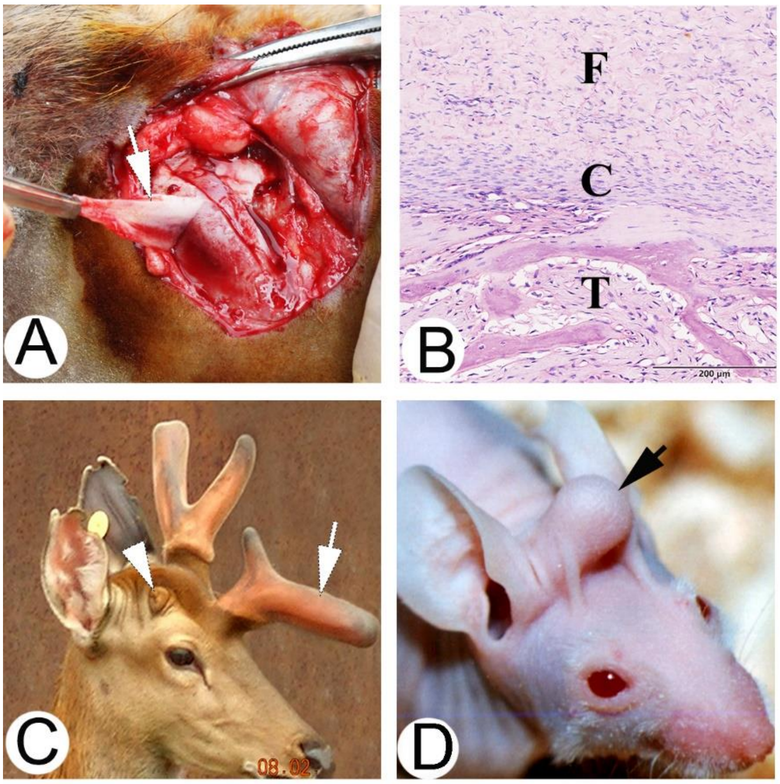

Figure S1. Establishment of deer antler autonomous self-differentiation system. (A) Sampling of antlerogenic periosteum (AP; arrow). (B) Histology of AP (F, fibrous layer; C, cellular layer; T, trabecular bone). (C) AP deletion and transplantation. Note that pedicle and antler failed to develop from the original site following the AP removal (arrowhead); whereas, an ectopic pedicle and branched antler developed from the AP grafted site (arrow; forehead). (D) Xenogeneic antler (arrow) developed from the grafted AP on the nude mouse head.

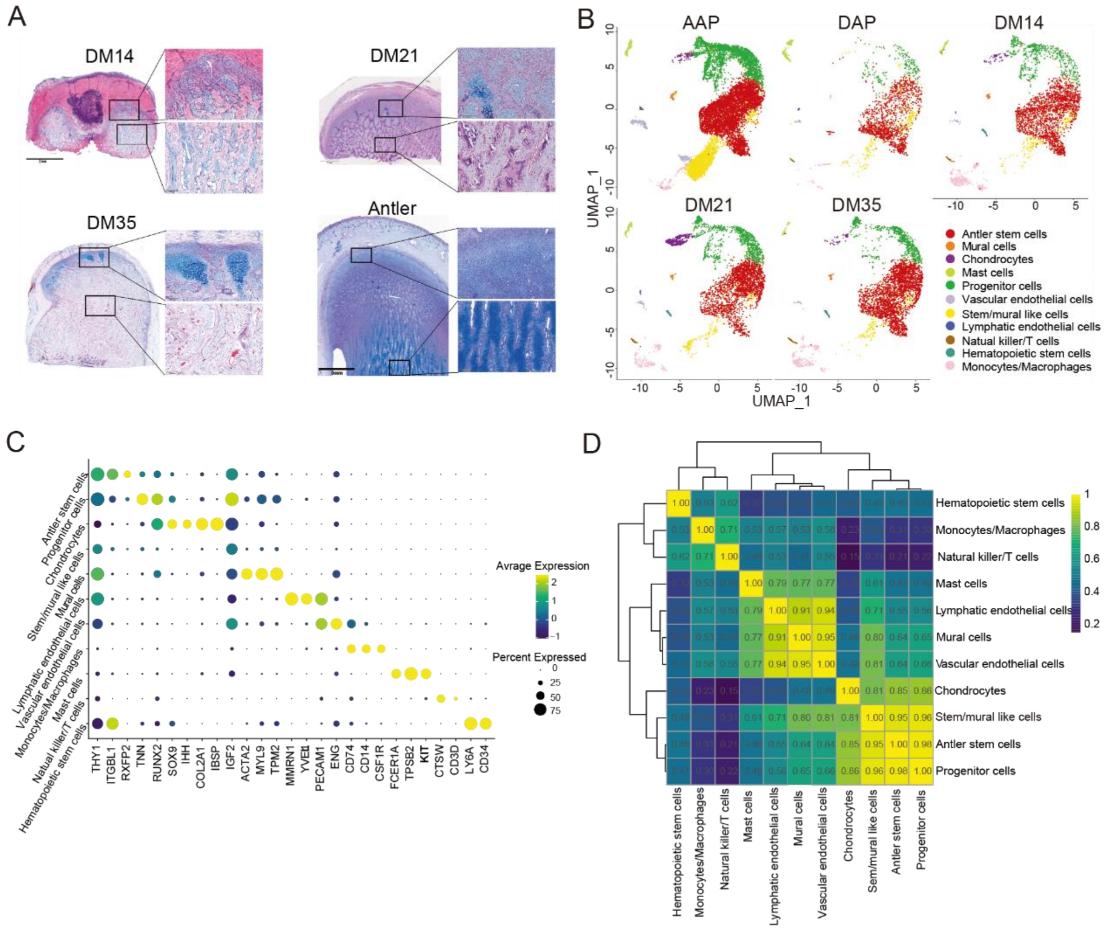

Figure S2. (A) Representative of histological sections of DMs/antlers (HE/Alcian staining). All tissue sections consisted of mesenchymal layer and cartilage, and more chondrocytes observed in the natural antler. (B) The UMAP of cell types in each tissue type (DAP, AAP, DM14, 21, and 35). (C) The dot plot of the marker genes corresponding to different cell types. Dot size: percentage of cells expressed the marker; and dot color: indicated the expression level of genes. (D) Inter-cell type correlation, calculated based on single-cell transcriptome data.

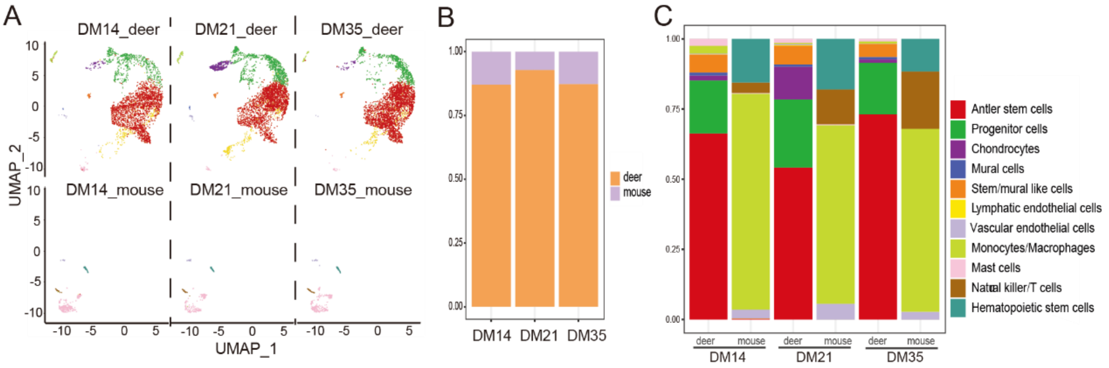

Figure S3. (A) UMAP of the cell composition in different tissue types. (B) Proportions of VECs of different origin in the xenogeneic antlers (DM14, DM21, DM35). (C) Proportions of different cell types in each tissue type.

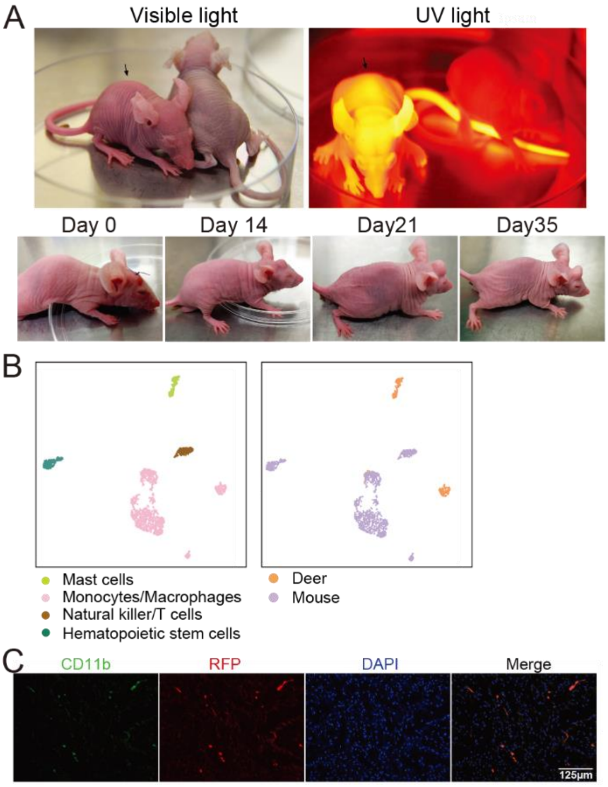

Figure S4. (A) Red fluorescent nude mice used for visualization of mouse derived cells in DMs. (B) UMAP diagram of immune cells in scRNA-seq: left panel, different cell types; right panel, species origin. Note that monocytes/macrophages, natural killer/T cells and hematopoietic stem cells (HSCs) were mainly derived from mouse, while mast cells were deer derivative. (C) Representative images of immunofluorescence staining demonstrated macrophages were mainly mouse derivative in DM21.

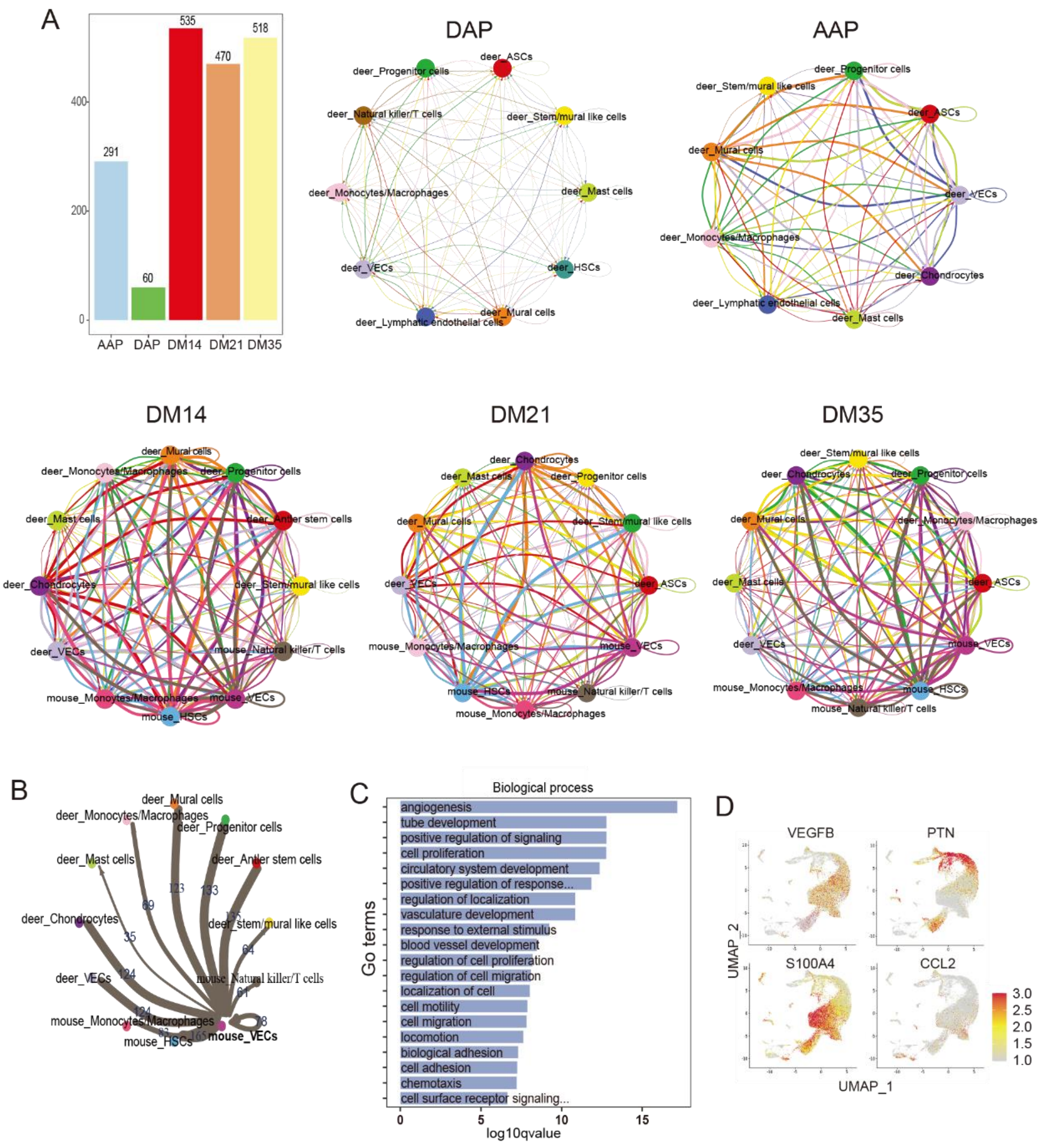

Figure S5. (A) Cell interaction results of different tissue types. The left panel: histogram of the cell interaction entries of different tissue types, and the second to sixth panels: network diagrams of the cell interaction results of each tissue type (DAP, AAP, DM14, DM21, and DM35). (B) Shell plot of the interactions mouse-derived VECs with other cell types in the DM14. Line thickness: number of interaction items. (C) GO enrichment analysis results of the interaction relationship. (D) UMAP diagram of the expression profiles of cytokines in all detected cell types.

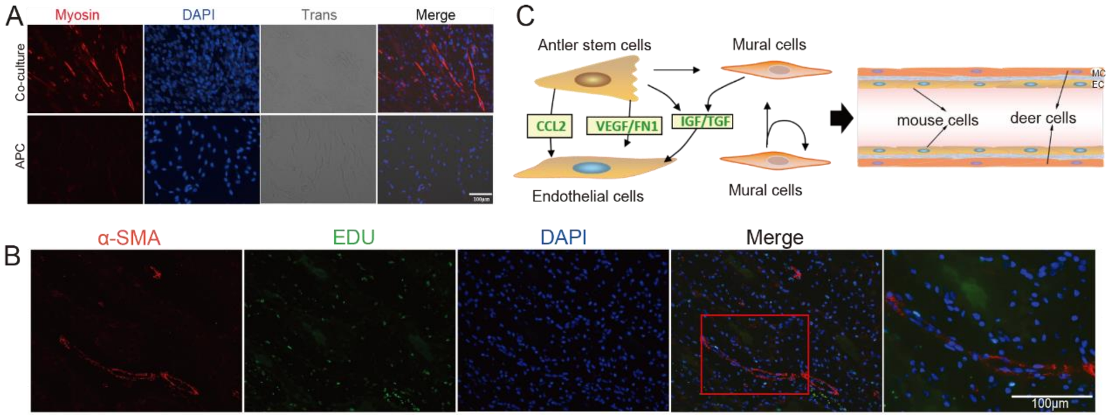

Figure S6. (A) Immunofluorescent staining of Myosin in the differentiated cells in the co-culture of APC and C2C12 for 10 days. (B) Double immunofluorescence staining showed that mural cells (α-SMA; red) consisted of proliferating cells (EdU; green). (C) Schematic drawing of the formation of chimeric vessels in the DMs.

Table S1. Antibodies used in this study

Terms	Manufacturer and product code	Application
Mouse anti-Myosin	Abcam; ab37484	IF, 1:500
Rabbit anti-TPSB2	ABclonal; A19801	IF, 1:200
Rabbit anti-CD34	Abcam; ab81289	IF, 1:100
Rabbit Anti-mCherry	Abcam; ab213511	IF, 1:200
Mouse Anti-mCherry	Abcam; ab125096	IF, 1:200
Rabbit anti-SCA1	Abcam; ab255604	IF, 1:200
Mouse anti-SMA	Abcam; ab7817	IF, 1:100, IHC, 1:500
Rabbit anti-CD31	Bioss; bs-0468R	IF, 1:50
Rabbit anti-CD14	Bioss; bs1192R	IF, 1:100
Rat anti-CD14 APC-Cy7	BD; 560636	Flow, 1:50
Rat anti-CD11b Alexa Fluor® 488	BD, 557672	IF, 1:50
Rabbit anti-Runx2	Abcam; ab236639	IHC, IF
Rabbit IgG-Isotype control	Abcam, ab172730	IF, Flow, dependent
Mouse IgG-Isotype control	Abcam, ab37355	IF, Flow, dependent
Goat Anti-Rabbit IgG H&L (Cy3 ®)	Abcam; ab6939	IF, Flow, 1:500
Goat Anti-Mouse IgG H&L (Cy3 ®)	Abcam; ab97035	IF, Flow, 1:500
HRP-conjugated goat anti-mouse IgG	Beyotime; A0216	IHC, 1:1000
HRP-conjugated goat anti-rabbit IgG	Beyotime; A0208	IHC, 1:1000
Goat anti-rabbit IgG H&L (Alexa Fluor 488)	Abcam; ab150077	IF, Flow, 1:500
Goat Anti-mouse IgG H&L (Alexa Fluor 488)	Abcam; 150113	IF, Flow, 1:500

[1]WB, Western-blot; [2]IF, Immunofluorescence; [3]Flow, Flow cytometry
